# Supplementary material for: Eliminating elevated p53 signaling fails to rescue skeletal muscle defects or extend survival in lamin A/C-deficient mice
Source: Cell Death Discov. 2024 May 22;10:245. doi: 10.1038/s41420-024-01998-1 (PMC11111808; doi:10.1038/s41420-024-01998-1)
Supplement: Supplementary file 1 — Supplementary Figures 1–3 [file 41420_2024_1998_MOESM1_ESM.docx]

**Supplementary Figures**

**
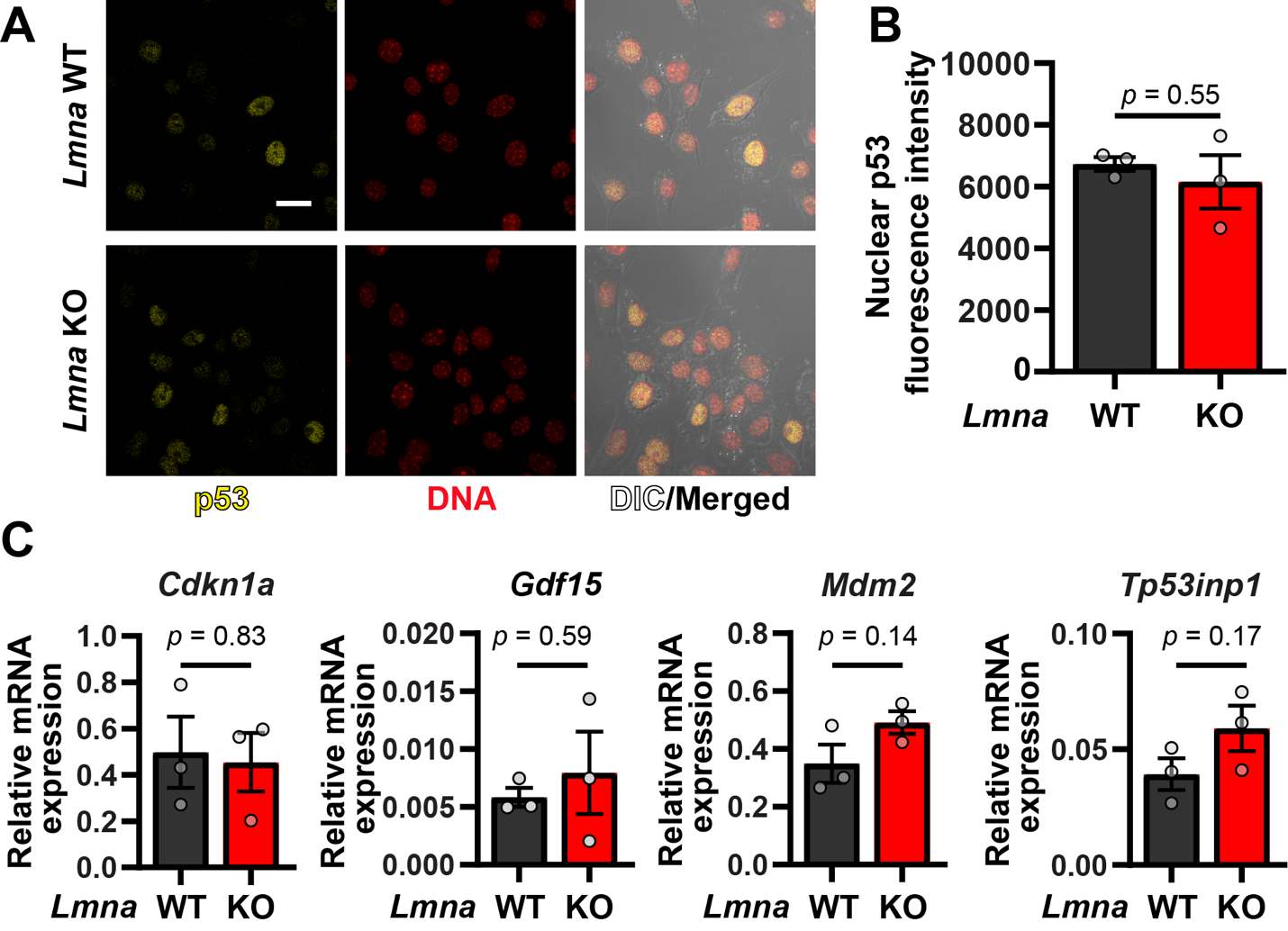
**

**Figure S1. p53 levels and p53-dependent genes are not differentially expressed in *Lmna* KO myoblasts prior to differentiation. (A)** Representative image of p53 immunofluorescence in primary myoblasts at day 0 of *in vitro* myofiber differentiation. Scale bar: 20 µm (**B**) Quantification of nuclear p53 immunofluorescence at day 0 of *in vitro* myofiber differentiation of *Lmna* WT and *Lmna* KO cells. (*N* = 3 independent cell lines with *n* = 83-158 nuclei quantified per genotype; Data shown as mean ± SEM; unpaired Student’s *t* test) (**C**) Quantification of relative gene expression of p53-dependent genes (*Cdkn1a*, *Tp53inp1*, *Mdm2* and *Gdf15*) at day 0 of *in vitro* myofiber differentiation of *Lmna* WT and *Lmna* KO cells. (*N* = 3 independent cell lines; Data shown as mean ± SEM; unpaired Student’s *t* test.)


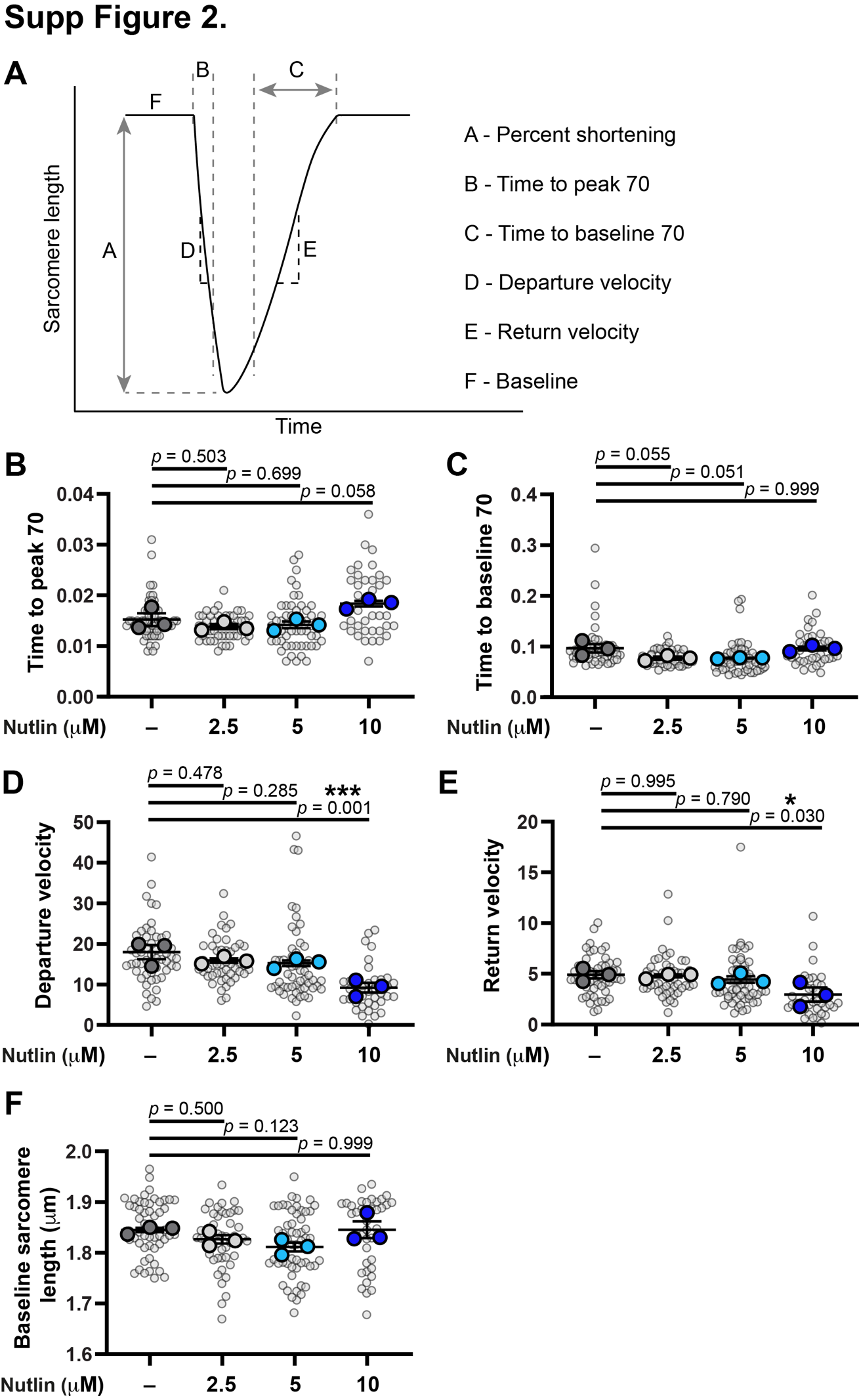


**Figure S2. p53 stabilization impairs muscle fiber contractility *ex vivo*. (A)** Representative contraction trace showing the contractile parameters measured using a high-throughput myocyte contraction system. **(B)** Quantification of the time required to reach 70% of peak sarcomere shortening (i.e. contraction time) in response to electrical stimulation in muscle fibers treated with different concentrations of nutlin-3 or DMSO control. (Data based on *N* = 3 mice and *n* = 38 – 55 muscle fibers; Data shown as mean ± SEM; One-way ANOVA with Dunnett’s post-hoc correction) **(C)** Quantification of the time to return from 70% of peak sarcomere shortening back to baseline (i.e., relaxation time) in response to electrical stimulation in muscle fibers treated with different concentrations of nutlin-3 or DMSO control. (Data based on *N* = 3 mice and *n* = 38 – 55 muscle fibers; Data shown as mean ± SEM; One-way ANOVA with Dunnett’s post-hoc correction) **(D)** Quantification of departure velocity (i.e., contraction velocity) in response to electrical stimulation in muscle fibers treated with different concentrations of nutlin-3 or DMSO control. (Data based on *N* = 3 mice and *n* = 38 – 55 muscle fibers; Data shown as mean ± SEM; One-way ANOVA with Dunnett’s post-hoc correction) **(E)** Quantification of return velocity (i.e. relaxation velocity) in response to electrical stimulation in muscle fibers treated with different concentrations of nutlin-3 or DMSO control. (Data based on *N* = 3 mice and *n* = 38 – 55 muscle fibers; Data shown as mean ± SEM; One-way ANOVA with Dunnett’s post-hoc correction). **(F)** Quantification of resting sarcomere length in muscle fibers treated with different concentrations of nutlin-3 or DMSO control. (Data based on *N* = 3 mice and *n* = 38 – 55 muscle fibers; Data shown as mean ± SEM; One-way ANOVA with Dunnett’s post-hoc correction)

**
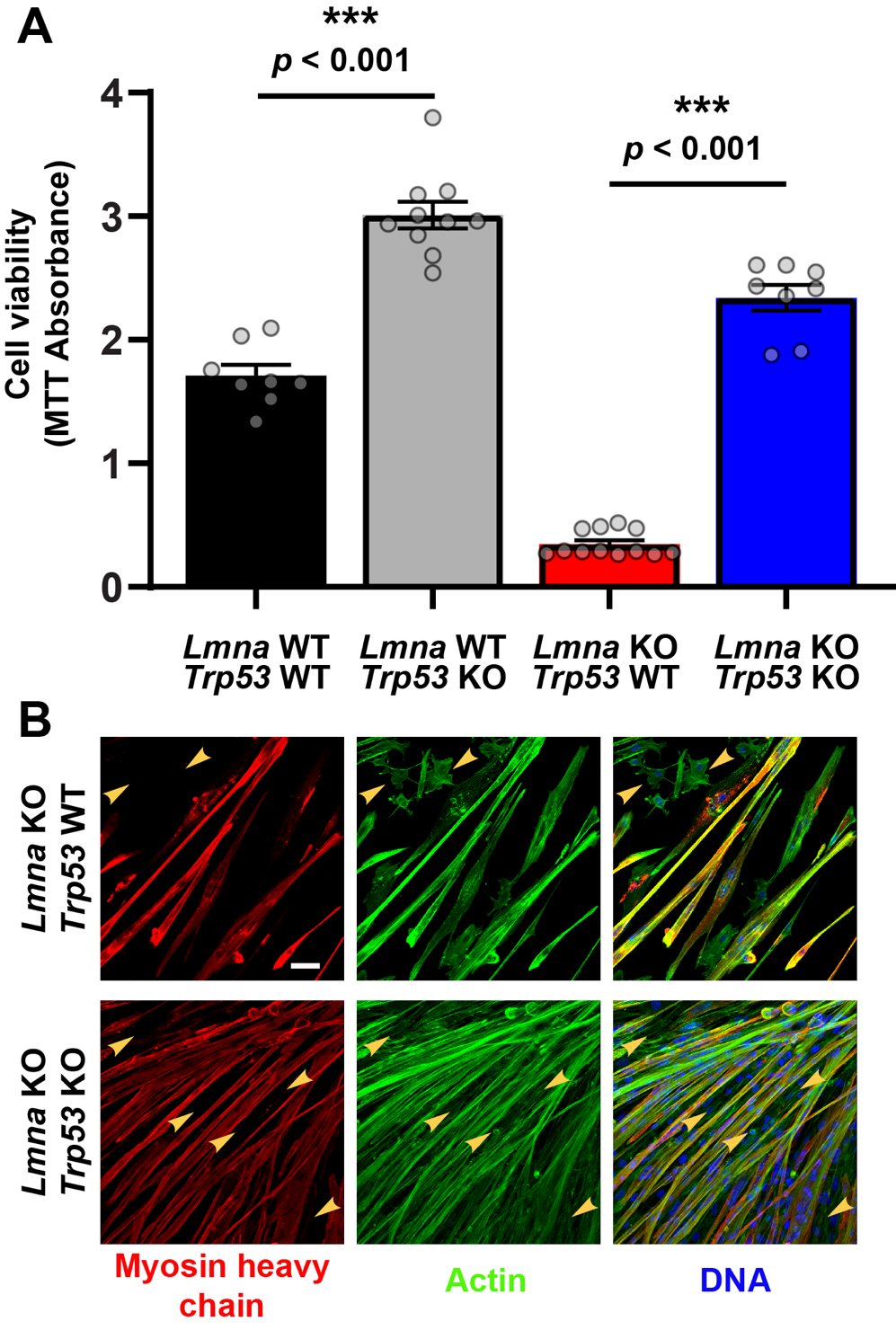
**

**Figure S3. Loss of p53 improves viability in *Lmna* KO muscle cells, but negatively affects myofiber differentiation. (A)** Quantification of myofiber viability using the MTT assay. (*N* = independent replicates from *n* = 3 independent cell lines; Data shown as mean ± SEM; unpaired Student’s *t* test) **(B)** Representative images of *Lmna* KO myofibers, either with (*Trp53* WT) or without p53 (*Trp53* KO) expression, at day 10 of differentiation. Arrows denote the presence of undifferentiated (Myosin heavy chain negative) cells, which increases with the loss of p53 expression. Scale bar: 50 µm.
